# Growth Curves and Predictors of Touch‐Typing Fluency in 12–13‐Year‐Old Students With and Without Dyslexia

**DOI:** 10.1002/dys.70053

**Published:** 2026-11-03

**Authors:** Janja Košir, Andrej Košir, Anja Podlesek

**Affiliations:** ^1^ Department of Special and Rehabilitation Pedagogy, Faculty of Education University of Ljubljana Ljubljana Slovenia; ^2^ The User‐Adapted Communications and Ambient Intelligence Lab, Faculty of Electrical Engineering University of Ljubljana Ljubljana Slovenia; ^3^ Department of Psychology, Faculty of Arts University of Ljubljana Ljubljana Slovenia

**Keywords:** dyslexia, touch‐typing fluency, transcription

## Abstract

Students with dyslexia often have difficulties with higher and lower‐level writing skills. Research shows that they have lower transcription speed and accuracy compared to their peers. A commonly recommended educational adjustment for addressing writing difficulties of these students is the use of a computer and typing instead of handwriting. However, there is evidence that students with language‐based handwriting problems can also type slowly. As dyslexia is also associated with difficulties in procedural learning, these students may have difficulties in learning touch‐typing. This study investigated how Slovenian students aged 12–13 years with dyslexia (*n* = 21) learn touch‐typing on a keyboard home‐row compared to their peers without dyslexia (*n* = 21). The growth curves for typing fluency were analysed using linear mixed modelling. The results showed that students with dyslexia achieved significantly lower typing fluency and slower learning progress at all learning stages. Students without dyslexia reached a learning plateau earlier. The achieved touch‐typing fluency was best predicted by simple finger typing fluency, pre‐existing typing technique fluency, and non‐word decoding fluency. Our study enhances understanding of the touch‐typing acquisition in students with dyslexia, though further research is needed to explore how this development unfolds across all phases of learning touch‐typing.

## Practitioner Points

1. Students with dyslexia acquire touch‐typing fluency more slowly than their typically developing peers, requiring individualised practice and extended learning time from the outset.
2. Touch‐typing may not be automatically easier than handwriting for students with dyslexia; assess individual needs before making substitutions.
3. Simple finger typing task fluency and self‐taught typing fluency can help identify students needing extra support in learning initial touch‐typing.

## 1 Introduction

### 1.1 Typing in Computer Writing

To address writing challenges experienced by students with dyslexia, computer typing has long been recognised as an effective means of support across various aspects of writing, e.g., in terms of fluency of writing, assistance with spelling, and revising and editing (Beers et al. 2017; Weigelt‐Marom and Weintraub 2016).

Typing requires the writer to memorise and recall a sequence of letters, locate the corresponding keys on the keyboard, and press them in the correct order with the appropriate fingers (Logan et al. 2016). Like handwriting, it is a fine‐motor, language‐based procedural skill (Cerni and Job 2024) that relies on both cognitive and sensorimotor processes (Mangen and Velay 2010); it requires retrieving target characters and maintaining information in memory (Feng et al. 2019). However, unlike handwriting which is based on the graphomotor formation of letters, typing requires successful recognition and selection of letters on the keyboard and the use of motor patterns for key presses (Preminger et al. 2004). When automated, it is therefore simpler in terms of the motor movement execution required for individual character output, as it does not require graphomotor formation parameters such as stroke type, correct stroke movements, and order for producing a single character. Nonetheless, unlike handwriting, which is typically unimanual, skilled typing requires effective bimanual coordination and the use of all fingers while maintaining visual attention on the computer screen rather than on the fingers (Crump and Logan 2010; Van Waes et al. 2017). In skilled typing, the motor execution space is separated from the visual space in which the text appears, making typing a sophisticated example of spatial ability that develops through a complex form of spatial learning (Mangen and Velay 2010).

The benefits of typing on a computer can only be fully realised when the skill is fluent and automated (Beers et al. 2017). Automatic typing skill evolves through three general phases of procedural learning: a cognitive phase marked by rapid improvement, a slower associative phase, and a final automation phase of procedural learning, where performance plateaus at a high level (Hong et al. 2019). With practice, typing can thus become an automated motor skill—performed quickly, accurately, and effortlessly—such that conscious control and attention to the fingers on the keyboard are no longer required (Logan et al. 2016), the intervals between keystrokes are short, and typists generally initiate the next key press before releasing the previous one (Dhakal et al. 2018). The main benefit of automatized typing is the reduction of the cognitive load on working memory, enabling writers to shift their focus from motor execution to higher‐order cognitive demands, such as generating ideas and organising content (Ahmed et al. 2021; Logan et al. 2016).

Typing can be learned either unsystematically—through incidental use and self‐directed practice—or systematically, through structured instruction (Dhakal et al. 2018). One of the most widely adopted systematic methods is the QWERTY keyboard touch‐typing technique, which emphasises the coordinated use of both hands, the engagement of all ten fingers, and the ability to type while maintaining visual focus on the computer screen rather than the keyboard. Some educational systems explicitly include typing instruction, such as in parts of the United States (Donica et al. 2018; Poole and Preciado 2016; Stevenson and Just 2014), Finland (Finnish National Agency for Education 2016), Switzerland (D‐EDK, Lehrplan 21 2014), Norway, and England (Pinet et al. 2022), although implementation is not always clear. In Slovenia, ICT is integrated across subjects in the primary school curriculum (ages 6–15), but fluent typing is not formally emphasised (Košir et al. 2024), and ICT use is below the OECD average (Ainley and Carstens 2018).

In general, research consistently shows that systematic instruction in typing leads to improved fluency and accuracy in typing in both younger (Christensen 2004; Donica et al. 2021; Van Weerdenburg et al. 2018) and older students (Weigelt‐Marom and Weintraub 2015). Christensen (2004) reported that 13‐year‐old students with low typing proficiency improved their typing effectiveness (number of letters typed in the correct alphabetical sequence within one minute) after eight weeks of a systematic typing skills programme practised for 20 min a day. Donica et al. (2021) found that all elementary school students in grades 2–4 who received weekly typing instruction over two years showed significant improvements in typing speed (words per minute), typing accuracy, and typing technique (assessed on a five‐level scale) when completing one‐minute and two‐minute Typing Test Pro keyboarding exercises. Van Weerdenburg et al. (2018) concluded that a touch‐typing course had a positive effect for students in grades 4–6 on their typing skills in a 10‐min text retyping task (improved keystrokes per minute, decreased typing errors), as well as on their spelling accuracy and narrative writing (number of words, description quality) when writing with a computer. Weigelt‐Marom and Weintraub (2015) reported improved typing fluency (average number of characters per minute) in typically achieving higher education students on a post‐test, three months after completing a touch‐typing instruction programme.

### 1.2 Typing Fluency in Students With Dyslexia and Its Factors

Among typically developing students, transcription skills including spelling and handwriting skills (Alves and Limpo 2015) as well as typing skills (Beers et al. 2017; Horne et al. 2011) generally become more automatic with age and experience. This developmental progression is evident in shorter pauses during writing, longer language bursts, and overall improvements in writing fluency (Alves et al. 2008; Alves and Limpo 2015; Beers et al. 2017).

For individuals with dyslexia, spelling and handwriting fluency are well‐documented areas of difficulty (e.g., Barnett et al. 2020; Beers et al. 2017; Graham et al. 2021; Van Heuverswyn et al. 2024; Košir et al. 2024). In contrast, evidence on typing skills or typing fluency in individuals with dyslexia remains comparatively limited, particularly among students at ISCED levels 1 and 2, as noted in several recent studies (Abecassis et al. 2023; Barnett and Stuart 2024; Jung et al. 2021; Košir et al. 2024; Thompson et al. 2017). Illustrating this gap, Barnett and Stuart (2024) identified only eight studies published between 2008 and 2024 that examined typing skills in school‐aged students (aged 4–17 years) with developmental disorders across languages, of these, only two included students with specific learning disorders involving reading difficulties or dyslexia. Studies on typing fluency in individuals with dyslexia also vary widely in their design and methods, which limits direct comparability of results. In light of these limitations, the following section adopts a descriptive approach to synthesising the literature, with a particular focus on observed patterns of typing fluency and typing technique in students with dyslexia.

Reduced typing fluency in individuals with dyslexia has been reported across a range of language‐based writing tasks. During expressive writing, lower typing fluency relative to typically developing peers was observed in 15‐year‐old Swedish students with specific reading and writing difficulties, with fluency measured using inter‐keystroke transition times and number of words produced (Wengelin et al. 2014). In a separate study of composed text generation, U.S. students with dyslexia in Grades 4–9 showed lower typing fluency and accuracy than peers during text composing, as quantified by words per minute and words per burst (Beers et al. 2017). Reduced typing fluency has also been documented in copy‐typing tasks. In this context, Hebrew‐speaking university students with specific learning disorders (reading or handwriting difficulties), demonstrated lower typing fluency than typically developing peers when fluency was measured as characters per minute (Abecassis et al. 2023), and 12–13‐year‐old Slovenian students with dyslexia showed lower text copy‐typing fluency than peers in correct characters per minute (Košir et al. 2024). In contrast, in a study of German students with dyslexia (mean age = 11.45 years), Jung et al. (2021) found that typing speed when copy‐typing and typing to dictation of words and pseudowords did not differ between students with dyslexia and typically developing peers. Japanese students with dyslexia aged 7–18 showed slightly lower typing fluency (number of letters or words) and accuracy than their peers without dyslexia when copying hiragana letters, and when typing meaningful and nonsense words, both by reading (copying) and from dictation; however, these differences were not statistically significant. Typing fluency increased with age in both groups. Students with dyslexia experienced fewer difficulties in dictation (listening‐based) typing tasks (Ishitani et al. 2024). In tasks with minimal or no linguistic writing context, fluency findings are inconsistent across studies. No group differences were observed in a speeded alphabet‐generation task (Beers et al. 2017), whereas reduced fluency was reported in a one‐handed fast sequential finger‐typing task involving an unfamiliar fine‐motor pattern (Košir et al. 2024).

Research on typing technique in individuals with dyslexia has primarily relied on observations from text composing and copy‐typing tasks, examining key features linked to efficient ten‐finger touch typing, including finger use and screen gaze.

In composing tasks, Beers et al. (2017) investigated U.S. students with dyslexia in Grades 4–9 and reported a predominance of visually controlled typing behaviour. Their findings indicated frequent shifts of visual attention toward the hands during text generation, rather than sustained visual monitoring of the on‐screen text, suggesting limited automatisation of typing skills. In copy‐typing tasks, similar patterns have been documented. Abecassis et al. (2023) examined Hebrew‐speaking university students with specific reading and/or writing learning disorders and assessed typing technique using screen‐gaze measures. Participants spent a relatively limited amount of time visually attending to the screen during copy typing, indicating reliance on keyboard‐directed visual guidance, contrasting with the screen‐based visual control characteristic of skilled touch typists. Likewise, Košir et al. (2024) reported that 12–13‐year‐old Slovenian students with dyslexia relied predominantly on visually guided typing and restricted finger use during text copy‐typing tasks, reflecting non‐automatised, self‐developed typing techniques rather than systematic ten‐finger touch typing. Weigelt‐Marom and Weintraub (2015) reported that most higher‐education students relied on visually guided, non‐automatised typing strategies prior to touch‐typing instruction, with few demonstrating efficient ten‐finger touch typing. Within the group of students with learning disabilities, however, a larger proportion (67%) typed in a particularly inefficient manner, most commonly using only one finger on each hand, compared to students without learning disabilities.

Taken together, the consistently observed lower typing fluency, compared to peers, of students with dyslexia in language‐based writing contexts (e.g., Wengelin et al. 2014; Beers et al. 2017; Jung et al. 2021; Abecassis et al. 2023; Košir et al. 2024) and also not fullly efficient typing technique raises the question of whether reduced typing fluency performance reflects limitations in typing technique, persistent language‐processing constraints, or an interaction of both. Lower typing fluency and less efficient typing technique tend to co‐occur in individuals with dyslexia.

These observations align with earlier findings reported by Bisschop et al. (2017) and Rosenberg‐Adler and Weintraub (2020), showing that students with language‐related difficulties may also struggle when writing by typing. Previous research has shown that typing fluency is related to several cognitive functions, including linguistic abilities and skills such as phonological encoding, decoding, and orthographic knowledge (Abecassis et al. 2023; Rosenberg‐Adler and Weintraub 2020), as well as reading speed (Gahshan‐Haddad and Weintraub 2023; Khoury‐Shaheen and Weintraub 2023; Malpique et al. 2023; Preminger et al. 2004). Typing, like this, relies on linguistic processes—particularly phonological and orthographic processing—which constitute core areas of difficulty in dyslexia. Consequently, students who experience reduced writing speed due to language‐processing constraints may write slowly both when handwriting and when typing.

Studies consistently report that students with dyslexia typing techniques deviate from the characteristics of efficient ten‐finger touch typing, including reliance on a limited number of fingers and predominantly visually guided typing with reduced attention to the screen (Wengelin et al. 2014; Beers et al. 2017; Abecassis et al. 2023; Košir et al. 2024). Beyond linguistic constraints, aspects of motor learning and automatisation of efficient typing technique may also contribute to reduced typing fluency in students with dyslexia, likely interacting with language‐processing demands during typing. Typing depends on the gradual proceduralisation of skill. As such, the co‐occurrence of reduced typing fluency and non‐automatised typing technique in students with dyslexia may be understood within a broader procedural learning framework. Research suggests that individuals with dyslexia may experience difficulties transitioning from declarative to procedural (automatic) performance (Nicolson and Fawcett 2018), with reported challenges across linguistic (e.g., Gabay et al. 2012) and motor domains (e.g., Denckla 1985; Lum et al. 2013; Nicolson and Fawcett 1990; Wolff 2002; Yap and van der Leij 1994). Evidence suggests that students with dyslexia may experience difficulties in acquiring and automatising complex sensorimotor skills (Marchand‐Krynski et al. 2017; Nicolson and Fawcett 2000). In direct relation to typing, empirical studies have shown that motor‐related variables such as fine motor typing skills—which are not primarily linguistic in nature—are associated with typing fluency, which is typically lower in students with dyslexia compared to their typically developing peers (Košir et al. 2024). Similar difficulties have also been observed in tasks as well as in learning procedural tasks that combine motor and linguistic components (Gabay et al. 2012; Nicolson et al. 2010). From this perspective, slower typing may partly reflect broader procedural‐learning constraints that hinder the automatisation of integrated motor–linguistic skills across transcription modalities. These constraints may be further exacerbated by impairments in executive functions, including attentional control and working memory, which are argued to interact with deficits in automaticity and procedural learning in dyslexia (Smith‐Spark and Gordon 2022).

### 1.3 Touch‐Tpying Learning in Students With Dyslexia

Despite the relevance of effective typing for students with dyslexia—characterised by fluency and reliance on kinaesthetic rather than visual feedback—research on typing development and learning in this population remains scarce. Fundamental questions therefore remain unanswered, including whether and to what extent typing fluency and typing technique can improve through systematic training in the presence of persistent language‐processing difficulties. Studies that explicitly examine typing instruction (e.g.,) touch typing in individuals with dyslexia remain isolated, offering only limited insight into how typing technique and typing fluency develop as learning processes in this population.

We identified only two studies that have explicitly examined the acquisition of ten‐finger touch typing as a learning process in individuals with dyslexia. One investigated Israeli university students with and without specific reading and/or writing disabilities using a structured, multi‐session instructional programme covering the full keyboard (Weigelt‐Marom and Weintraub 2015; Weigelt‐Marom and Weintraub 2016; Weigelt‐Marom and Weintraub 2018), while the other, reported in our earlier analysis, examined 12–13‐year‐old students with dyslexia using a developmentally constrained training design focused on home‐row characters (Košir et al. 2024). Although both studies addressed typing acquisition, they differed markedly in developmental context, instructional scope, and analytic emphasis, providing complementary perspectives on learning processes.

In the university‐level study, Weigelt‐Marom and Weintraub reported that typing speed increased following instruction in both groups, and that initial group differences—students with learning disabilities typing more slowly than peers—were no longer statistically significant at delayed post‐test, suggesting successful acquisition of touch‐typing skills in adulthood. In contrast, Košir et al. (2024), who focused on the early stages of typing learning in younger students, report that at the baseline, students with dyslexia demonstrated significantly lower fluency when using self‐taught typing techniques in text‐copying tasks, and they remained less fluent than peers during and at the end of touch‐typing training restricted to home‐row characters. Importantly, authors reported a statistically significant positive learning trend in typing fluency also in students with dyslexia, indicating measurable improvement over the course of instruction, despite lower absolute performance levels compared to typically developing peers.

Methodologically, Weigelt‐Marom and Weintraub evaluated longer‐term acquisition following extended, full‐keyboard instruction, whereas Košir et al. (2024) focused on early learning dynamics using repeated measurements during short‐term, home‐row‐restricted training.

These two studies suggest that individuals with dyslexia do make progress in typing fluency and typing technique through instruction; however, it remains unclear how large these gains are and how the pace and stability of improvement depend on developmental stage and stage of practice or instructional scope. Critically, apart from these two investigations, no other studies have systematically examined how touch‐typing skills are learned in individuals with dyslexia, nor have any research addressed how different dyslexia‐specific factors (cognitive, linguistic, motor) jointly shape learning trajectories in students with dyslexia. Given that students with dyslexia are widely encouraged to use computers for writing, the lack of empirical evidence on how touch typing should be taught, how automatisation unfolds, and which factors hinder or facilitate successful learning represents a significant gap in the literature. Addressing the learning process of touch typing—rather than solely its performance outcomes—is therefore essential for developing evidence‐based recommendations for instructional practice in this population.

### 1.4 Research Design and Aim

In our previous analysis (Košir et al. 2024), we found differences in touch‐typing learning fluency and identified differences between students with dyslexia and their typically developing peers on selected measures of cognitive, linguistic, and fine motor skills, as well as in handwriting fluency. Building on these findings, the present paper extends our earlier work—which compared only initial and final typing fluencies and implicitly assumed linear improvement—by following both student groups throughout a structured touch‐typing training period. Specifically, we examined the learning trajectories of typing fluency and investigated the extent to which these trajectories, as well as final touch‐typing fluency outcomes, are associated with selected cognitive, linguistic, and fine motor abilities as potential contributors to touch‐typing acquisition and performance.

In this paper, we hypothesised that individual touch‐typing learning trajectories and final touch‐typing outcomes would be associated with cognitive, linguistic, and fine motor abilities, and that students with dyslexia would exhibit learning trajectories that differ from those of their typically developing peers.

We used a two‐group longitudinal repeated measures design with two age‐matched between‐subjects groups: students with dyslexia and typically developing peers. Participants were assessed multiple times during the touch‐typing training.

The study was approved by the Ethical Committee of the Faculty of Education, University of Ljubljana, Slovenia.

## 2 Methods

### 2.1 Participants

Information about a two‐week touch‐typing course was sent to 46 primary schools in Ljubljana, the Faculty of Education at the University of Ljubljana, to The Counselling Centre for Children, Adolescents and Parents in Ljubljana, and published on a website and a Facebook page. Students were recruited through voluntary participation and targeted selection. Parents and students received information from teachers and special education teachers at their primary schools, as well as from professionals at the counselling centre and directly from the researchers at the Faculty of Education. They then contacted the first author of this study to confirm whether they met the inclusion criteria, and upon successful verification, received confirmation of their eligibility to participate.

Participants were 7th‐ or 8th‐grade students who had no previous experience with touch‐typing. Students without dyslexia were included only if parents confirmed that their child had no diagnosed learning difficulties, no other special needs, and had not been identified as gifted. Parents of students with dyslexia had to present an official state‐issued certificate for a specific reading disorder, based on prior in‐depth expert assessments. All certificates were reviewed by the first author, a special education teacher. The identification procedure in Slovenia includes comprehensive cognitive testing for dyslexia indicators (e.g., phonological awareness, rapid naming, reading, spelling, working memory and intelligence tests) as well as questionnaires and interviews with teachers, children and parents and the analysis of students' work samples and reading/writing tests (Košak‐Babuder et al. 2019). The Slovenian criteria for a specific learning disorder in reading include an IQ performance discrepancy (IQ above 85) and cognitive processing deficits. All students with dyslexia in our sample had previously obtained official certification of their disability status through this procedure. Nineteen students in the dyslexia group also had co‐occurring writing difficulties, and attention deficits were reported for 12 of them but were not explicitly stated as ADHD.

All parents confirmed the inclusion criteria and provided consent for participation and data processing. All participating students were Slovenian, with Slovene as their mother tongue. All students had to agree to participate by signing a consent form.

All students had a home computer and internet access, which they used mainly for leisure and only occasionally for school‐related tasks.

Before conducting the experiment, we estimated the required sample size using an a priori power analysis. For the difference between groups (students with dyslexia and their typically developing peers), we assumed an effect size of 0.80 and a statistical power of 0.80, resulting in an estimated sample size for an one‐tailed *t*‐test of *n*_1_ = *n*_2_ = 21. As some drop‐out was expected, we recruited a total of 46 students. Of the 46 students who had completed the touch‐typing course, only 42 students, i.e., 21 from each group, had complete data due to technical issues. This exact balance between the groups was unplanned and occurred by chance. The final analysis thus included data from 21 students with developmental dyslexia (DD) and 21 typically developing peers without dyslexia (TD). The two groups did not differ statistically significantly in age (M_DD_ = 13.3 years, SD_DD_ = 0.63 and M_TD_ = 13.2 years, SD_TD_ = 0.56, *t*(40) = 0.08, *p* = 0.935). In the DD group, 13 students were males (ten 7th‐graders, three 8th‐graders) and 8 were females (four 7th‐graders, four 8th‐graders). In the TD group, 11 students were males (ten 7th‐graders, one 8th‐grader) and 10 were females (six 7th‐graders, four 8th‐graders). The final short touch‐typing fluency test results after the 11th interval of the third touch‐typing stage (see procedure under Table 1) was similar for both males and females (M = 136, SD = 31 for girls and M = 127.3, SD = 31.3 for boys, with the difference between genders not reaching statistical significance, *t*(33) = 0.793, *p* = 0.217).

**TABLE 1:** Two‐week procedure of daily touch‐typing training across three touch‐typing stages, with other test tasks administered before, between, and after the touch‐typing sessions.

Week	Monday	Tuesday	Wednesday		Thursday	Friday
1st	First stage (AFJČ)			Second stage (ADFJKČ)		
	TIIIF	IIII	ICF	III	IIII	CIIIFI
2nd	Third stage (ASDFJKLČ)					
	CIFIII	IIII	IIAFIII		IIII	IIFS

*Note:* A, touch‐typing test after the 11th 10‐min interval in the third stage; C, interval used for game‐based training in TypingClub.com, for which data were not available; F, a finger‐typing task; I, 10‐min touch‐typing learning interval; S, SNAP tests; T, test of handwriting and self‐taught typing fluency.

### 2.2 Measures

Apart from assessing touch‐typing fluency, we used a variety of measures to evaluate students' skills and abilities: a series of SNAP tasks to evaluate specific cognitive and linguistic skills, a simple finger‐typing task to assess fine motor skills, and tasks designed to assess pre‐training transcription skills, including handwriting and self‐taught typing fluency.

#### 2.2.1 Handwriting Fluency and Self‐Taught Typing Fluency

Before the touch‐typing training, we assessed students' handwriting fluency and their self‐taught typing fluency using a text‐copying task. Three age‐appropriate narrative prose texts without pictures or dialogues, which were similar in length and very easy or easy to read according to LIX readability formula (Björnsson 1968), were selected from an age‐appropriate literature textbook. Two non‐adjacent 100‐word passages in Arial 18 were selected from each text: one for handwriting and one for typing. This resulted in three passages for each task. The students transcribed each passage in succession, for one minute. For the typing task, our own developed touch‐typing application was used. Students could correct typing errors but did not have access to spell check or word prediction. They were instructed to transcribe quickly and accurately while, when handwriting, also maintaining legibility of handwriting. We assessed and compared the students' handwriting and self‐taught typing fluency by counting the number of correct characters per minute (CPM). All letters and punctuation marks were included as recommended by Weigelt‐Marom and Weintraub (2015), but spaces and capitalization were excluded following Feng et al. (2019). A more detailed description of this task can be found in Košir et al. (2024). The writing samples were first coded to ensure anonymity during assessment. Typing performance was scored automatically using character counts in Microsoft Word, while handwriting performance was scored by the first author, an experienced special education teacher, counting legible letters. Given the good legibility of individual letters and the established reliability of writing fluency measures in the literature (e.g., Rosenberg‐Adler and Weintraub 2020), no additional rater was employed.

The three self‐taught typing fluency and three handwriting fluency measures showed high internal consistency in the whole sample, using ICC (3,1). For typing fluency, ICC = 0.97, 95% CI [0.95, 0.98]; for handwriting fluency, ICC = 0.95, 95% CI [0.91, 0.97]. Results were similar in both subgroups: for students with dyslexia, typing fluency ICC = 0.94, 95% CI [0.88, 0.98], and for the control group, ICC = 0.96, 95% CI [0.92, 0.98]; handwriting fluency was the same in the dyslexia and control groups, ICC = 0.94, 95% CI [0.88, 0.97]. Inter‐item correlations were consistently high. For further analyses, we used the mean value across the three measurements.

#### 2.2.2 Simple Finger‐Typing Task

Fine motor fluency was assessed using a finger‐typing task (FTT), in which participants repeatedly typed the sequence 41,324 on a keyboard using the four fingers of their dominant hand (excluding the thumb), which is the hand they normally use for writing. This task was based on studies by Cellini (2017) and Biotteau et al. (2015), and was used in Košir et al. (2024) as a motor sequence learning task with minimal language demands. To avoid imposing additional memory load, the sequence remained visible on the screen throughout execution. The task was implemented in a customised typing application developed for this study (see Touch‐Typing Training section). The simple finger‐typing task was administered according to the guidelines provided by the study's first author. Students practised the sequence on site every other day and performed three trials per week, for a total of six trials (see Table 1). Each trial consisted of four 30‐s blocks in which the sequence was repeated, followed by a 20‐s pause. On each trial, the students were first tested individually for correct memorisation and motor repetition of the sequence 41,324 before the first practise block. Keystrokes were measured using a specially prepared task in our typing application. On the computer screen, participants saw a 30‐s timer for the repetition of the sequence and a 20‐s timer for the pauses. We observed them during the task but gave them no feedback. Mastery of the sequence was assessed as the simple finger typing task fluency, by the number of correct repetitions (CSR) in 30 s, following Cellini's (2017) method. For a more detailed description of this task, see Košir et al. (2024).

For the second to sixth learning trials, the fluency of the simple typing task showed high internal consistency, using ICC (3,1). Reliability was very high in the whole sample (ICC = 0.95, 95% CI [0.91, 0.97]), and separately in both subgroups: for students with dyslexia (ICC = 0.92, 95% CI [0.84, 0.97]) and for the control group (ICC = 0.95, 95% CI [0.89, 0.98]). Inter‐item correlations were moderate to high in all groups (*r* = 0.63 to *r* = 0.91), confirming stable consistency across the five time points. The sixth trial was used as the final result for the analysis in this paper.

#### 2.2.3 Special Needs Assessment Profile (SNAP)

After the touch‐typing training, the Slovenian version of the SNAP Special Needs Assessment Profile (Weedon and Reid 2008) was used to assess specific learnig disorders with impairment in reading. SNAP is a widely used and validated instrument for assessing dyslexia risk in Slovenia (e.g., Košak‐Babuder et al. 2019). We used the following tests from the battery: Timed Reading Test (the number of words read correctly in 30 s, SNAP RF), Phonological Awareness Test (a phoneme deletion task in which students have to delete certain phonemes in non‐words they hear, SNAP PHON), Non‐Word Reading Test (the number of non‐words read correctly in one minute, SNAP NW), Timed Spelling Test (the number of words spelt correctly in a two‐minute dictation, SNAP SPELL), Backward Span Test (the number of successful repetitions of the backward word sequence, SNAP WM) and Picture Naming Test (the seconds needed to name pictures, SNAP SN). The SNAP test was administered individually to all participants on the final day of the study, following a standardised procedure with standardised instructions. Three certified special education teachers, invited specifically for this purpose and not involved in the touch‐typing instruction, conducted the testing. All test administrators were blinded to the participants' group membership (dyslexia or control).

#### 2.2.4 Touch‐Typing Training

The students took part in a two‐week touch‐typing course on the middle row of a QWERTZ keyboard (ASDFJKLČ) during the 2020/2021 or 2021/2022 school holidays. Thirteen training groups (six in 2020/2021, seven in 2021/2022) were formed, each with two to six students, to ensure the quality of lessons. The first author conducted all touch‐typing lessons, drawing on insights from experienced touch‐typing teachers, relevant literature (Beaton 2012) and 30 h of prior formal training. The typing course was adapted from a Slovenian touch‐typing textbook (Degen 1997) and structured into progressive learning stages based on the gradual introduction of new keyboard characters. The lessons followed a fixed letter sequence—AFJČ in Stage 1, AFJČDK in Stage 2, and AFJČDKSL in Stage 3 of the training. All participants followed a structured protocol implemented uniformly across students, including sequence for letter introduction, uniform lesson duration, and teacher feedback on body posture, finger positioning, and screen gaze (only brief prompts without pausing, with corrective feedback and result analysis provided only at the end of each interval), up to the end of the 11th interval in Stage 3 and the final touch‐typing test, which constituted the analytic segment for which results were analysed. After this point, intervals in Stage 3 (12–20), as shown in the study procedure table, reflected a shift toward more individualised support also during, not just after 10‐min typing intervals (e.g., longer feedback, motivation, and technique correction). Although participants continued practising beyond interval 11, these data are reported for transparency but are not included in the main analysis due to lack of comparability across stages and students in instructional support after interval 11.

The touch‐typing lessons and their duration were determined based on prior experience with intensive touch‐typing training formats to ensure feasibility and successful completion for all participants within the available timeframe. An intensive touch‐typing learning format was deliberately chosen to minimise the impact of uncontrolled variables, such as unsupervised practice and additional computer use, thus ensuring a reliable assessment of the programme's impact. Summer scheduling was the only feasible option and limited the recruitment of participants for longer training periods involving a more extensive set of keyboard characters.

The touch‐typing training was conducted using a customised typing web application developed specifically for this study, which included touch‐typing lessons, a self‐taught typing fluency task, and a simple finger‐typing task. In touch‐typing lessons, students copied lesson texts displayed on the screen in Montserrat sans‐serif font on a light‐yellow background. Each line contained up to six words or pseudowords (maximum 40 characters per line). Correct keystrokes were highlighted in green, incorrect ones in red, and the next character was underlined in blue. Errors could not be corrected during practice. The application, developed in JavaScript with a MySQL database, was extensively tested prior to the study and designed to fulfil age‐appropriate, research‐specific and ethical data access requirements. Details can be found in Košir et al. (2024). All participants used the same model of external keyboard and accessed the program via the Chrome browser. At each typing stage, participants completed a single 10‐min supplementary gamified session on the TypingClub.com touch‐typing platform, allowing them to practise the same content lessons in a game‐like format alongside the primary training in our application. This session was intended to provide variety, helping maintain engagement throughout the training.

The training was conducted over 10 consecutive weekdays: on‐site at the Faculty of Education in Ljubljana, Slovenia on Mondays, Wednesdays, and Fridays (50 min each) and online via Zoom on Tuesdays and Thursdays (40 min each). Typing was practised in 10‐min intervals. New letters were added in each stage, whereby the ratio of new to already learnt letters was at least 6:1. Typing fluency was recorded in all 10‐min intervals via our web application. Correct keystrokes were highlighted in green and errors in red, but typing was not blocked to correct mistakes, and students did not correct errors during input. At the end of each interval, they received automated feedback on their typing speed (correct characters per minute, CPM) and accuracy (percentage correct) on the screen, along with teacher feedback on posture, finger placement, and screen gaze.

Fluency was calculated as correct characters per minute (CPM), excluding pauses of more than 3 s and repeated characters (more than 4 times). Repeated character sequences (e.g., “rrrr”) were removed to prevent distortions caused by prolonged key presses from disproportionately affecting accuracy and fluency scores. Periods of inactivity longer than 3 s were excluded to avoid their influence in calculating students' actual typing ability. These preprocessing steps were applied automatically using a custom Python script after the completion of testing.

### 2.3 Procedure

Table 1 presents the design of the touch‐typing training and testing procedures. All students completed the same sequence of tasks.

First, handwriting and self‐taught typing fluency tests were administered. Touch‐typing training then took place each day in 10‐min intervals over two weeks. During this period, the finger‐typing task was also administered six times (three times per week, every other day). Touch‐typing fluency during the 10‐min training intervals was recorded and analysed: intervals 1–8 in Stage 1, intervals 1–7 and 9–11 in Stage 2, and intervals 2–11 in Stage 3.

After the 11th 10‐min interval of Stage 3, a short touch‐typing test (marked with A in Table 1) was conducted in which students were asked to type 42 words and pseudowords (4–6 letters, 2–3 syllables) as quickly and accurately as possible. The set of words and pseudowords for this test was based on those used in the 10‐min intervals during the third stage of typing instruction. Each word appeared only once in the list. The sequence included all letters introduced during the learning process. Each word in the list contained at least one, and in some cases both, of the letters added at the third stage (S and L). The format of the test was the same as that of the touch‐typing lessons, which were in a copy‐text format.

Finally, SNAP tests were conducted. Despite some technical problems that led to a partial loss of keystroke data (6%–7.4% per stage of touch‐typing), all trainings and measurements went as planned.

## 3 Data Analysis

Raw data are available on the Zenodo data repository (Košir et al. 2025). The data were processed in MS Excel, SPSS 29, and Jamovi (version 2.3.28) with the GAMLj module.

Descriptive statistics for the SNAP tests, handwriting fluency test, simple finger‐typing fluency test, self‐taught typing fluency test, and final touch‐typing test were calculated for students with dyslexia and their typically developing peers and are presented in Table 2. Normality of frequency distributions of variables in each group of students was tested using the Kolmogorov–Smirnov test. Outliers were defined by Tukey's method in SPSS. The groups were compared using one‐tailed *t*‐tests or Mann–Whitney *U* tests in case of non‐normal distributions, with a 5% alpha error rate.

**TABLE 2:** Comparison between students with dyslexia (DD) and typically developing peers (TD) with outliers excluded.

Variable	DD			*p*	TD			*p*	Group comparison	
	*n*_1_	M	SD		*n*_2_	M	SD		*Statistics, p*	Effect size, 95% CI
SNAP NW	21	22.8	5.0	0.311	20	35	3.78	0.183	*t* = −8.78, *p* < 0.001	−2.74 [−3.60, −1.87]
SNAP RF	21	47.1	13.4	0.022	19	76.0	8.05	0.914	*U* = 2, *p* < 0.001	0.99
SNAP WM	20	8.05	1.73	0.017	21	9.48	2.09	0.530	*U* = 124, *p* = 0.012	0.41
SNAP SN	21	49	10.8	0.878	20	38.7	6.11	0.648	*t* = 3.79, *p* < 0.001	1.17 [0.50, 1.83]
SNAP SPELL	21	12	3.66	0.308	17	15.5	2.50	0.799	*t* = −3.38, *p* = 0.001	−1.10 [−1.78, −0.41]
SNAP PHON	21	8.76	1.18	0.002	21	9.43	0.76	< 0.001	*U* = 152, *p* = 0.032	0.31
STT	21	48.8	14.7	0.193	18	79.0	17.1	0.853	*t* = −5.93, *p* < 0.001	−1.90 [−2.66, −1.13]
FTT	19	13.5	4.59	0.345	19	17.4	3.59	0.636	*t* = −2.92, *p* = 0.003	−0.95 [−1.61, −0.27]
HW	21	67.6	18.7	0.192	19	81.6	15.4	0.889	*t* = −2.56, *p* = 0.007	−0.81 [−1.45, −0.16]
TF_1_	19	59.2	8.9	0.200	21	83.2	20.3	0.100	*t =* −*4.91, p* < 0.001	−1.50 [−2.2, −0.79]
TF_2_	18	114	24	0.339	16	147	25.2	0.714	*t* = −3.88, *p* < 0.001	−1.33 [−2.07, −0.58]

*Note:* Range of outliers excluded: 0–4 per group.

Abbreviations: HW, handwriting fluency; FTT, simple typing task fluency; SNAP NW, non‐word reading fluency; SNAP PHON, phonological awareness; SNAP RF, reading fluency; SNAP SN, speed in picture naming; SNAP SPELL, spelling; SNAP WM, verbal working memory; STT, self‐taught typing technique fluency; TF_1_, touch‐typing fluency at the beginning of learning touch typing—during the first 10‐min interval of touch‐typing learning; TF_2_, touch‐typing fluency test after the 11th 10‐min interval in the third stage.

The descriptive statistics for 10‐min touch‐typing intervals are shown in Table 3. To model the growth curves of touch‐typing fluency at each learning stage, we used linear mixed modelling (LMM). Touch‐typing intervals were recorded at consecutive time points (eight for Stage 1 and ten for Stages 2 and 3) to create Level 1 data, that is, repeated measurements collected over time within the same individual. These data were centred to reduce the correlation between parameter estimates (see Enders and Tofighi 2007).

**TABLE 3:** Sample size and means, and standard deviations in parentheses, of touch‐typing fluencies in characters per minute in 10‐min touch‐typing intervals for students with dyslexia (DD) and typically developing Peers (TD) with outliers excluded.

Interval	1st stage		2nd stage		3rd stage	
	DD	TD	DD	TD	DD	TD
1st	19, 59.2	21, 83.2	20, 83	21, 109	C	C
	(8.9)	(20.3)	(12)	(28)		
2nd	18, 72	21, 99	19, 83	21, 118	18, 83	19, 108
	(18)	(25)	(11)	(34)	(7)	(24)
3rd	18, 77.4	16, 109	18, 87.2	15, 123	18, 86	19, 116
	(17)	(32)	(12)	(22.4)	(9)	(24)
4th	17, 80	18, 118	16, 94	20, 134	19, 88	20, 129
	(7)	(18)	(9)	(26)	(13)	(30)
5th	17, 89	16, 124	17, 101	20, 142	17, 89	20, 136
	(9)	(20)	(13)	(27)	(10)	(40)
6th	17, 92.2	16, 132	14, 100	17, 147	16, 93	18, 139
	(8)	(22)	(14)	(23)	(12)	(28)
7th	14, 92	17, 135	16, 100	16, 149	17, 97	16, 144
	(7)	(20)	(10.2)	(23)	(18)	(33)
8th	18, 102	18, 149	C	C	16, 99	18, 146
	(20)	(25)			(11)	(34)
9th	C	C	19, 109	19, 158	16, 103	17, 144
			(19)	(25)	(16)	(34)
10th	/	/	20, 116	18, 163	17, 109	19, 157
			(26)	(26)	(27)	(38)
11th	/	/	19, 111	18, 161	16, 107	16, 161
			(23)	(24)	(12)	(38)

*Note:* Values are presented as *n*, M (SD). C, interval used for game‐based training in TypingClub.com, for which data were not available.

The trajectory was specified as follows:

$$\begin{array}{c} \text{Touch}-\text{typing fluency}\;\left(\text{correct characters}\;\mathrm{per}\;\text{minute}\right)=\\ b_{0}+b_{1}\times \left(\text{centred time}\right)+b_{2}\times \left(\text{centred}\;{\text{time}}^{2}\right)+\text{error}. \end{array}$$

The random‐effect linear term (*b*_1_) captured the rate of change in fluency over time, while the random‐effect quadratic term (*b*_2_) reflected the curvature of the trajectory, indicating whether the rate of change accelerated or decelerated over time. Student group (dyslexia vs. no dyslexia) was included as a fixed predictor of all three *b* parameters. Inspection of the data indicated that the assumptions for implementing the linear mixed model—low correlations between estimators, linear relationships between the dependent and fixed‐effects variables, homoscedasticity, and normally distributed residuals—were satisfactorily met. We present the results of growth curve modelling, excluding outliers in 10‐min typing intervals.

The Holm–Bonferroni correction (*m* = 11) was applied, and all differences between students with dyslexia and typically developing peers remained significant.

## 4 Results

Descriptive statistics for different measures and group comparisons are presented in Table 2, and descriptive statistics for 10‐min touch‐typing training intervals for the three touch‐typing learning stages are shown in Table 3.

Students with dyslexia scored lower than their typically developing peers on all SNAP tests. The groups differed on key dyslexia indicators, supporting the validity of the sample. As expected, students with dyslexia were also less fluent than their peers in the following tasks: text copying handwriting fluency, simple pattern finger typing task fluency, text copying self‐taught technique typing fluency, and the touch‐typing learning final result of touch‐typing fluency. This indicates a general disadvantage in transcription fluency for this group, extending beyond handwriting to typing, including non‐verbal motor pattern typing learning.

One of the primary goals of our study was to examine how the growth curves of touch‐typing fluency differed between students with dyslexia and their typically developing peers. Figure 1 displays mean touch‐typing fluency for both groups, with and without outliers, across each stage of the touch‐typing learning process. As shown, students without dyslexia generally achieved higher average touch‐typing fluency than students with dyslexia. In both groups, fluency declined when new letters were introduced at the transition to a new stage. Moreover, the average growth curves in Stages 1 and 2 were slightly steeper and more decelerated compared to Stage 3.

**FIGURE 1:**
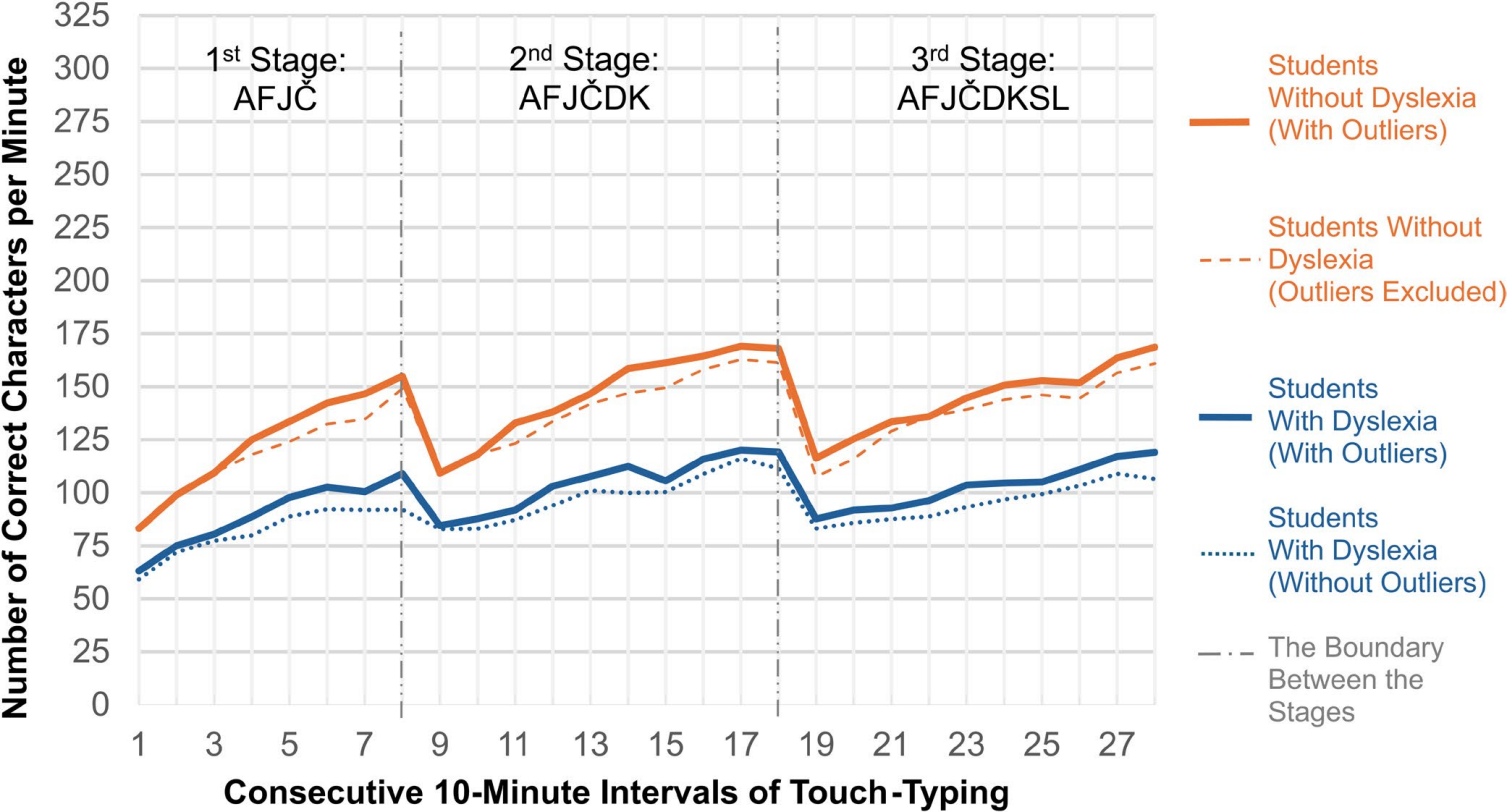
Growth curves of typing fluency across the three stages of touch‐typing learning in students with dyslexia and their typically developing peers.

The LMM analysis revealed that, for Stages 1 and 2, a model including both linear and quadratic terms for the growth curve provided an adequate fit. In contrast, for Stage 3, the inclusion of the quadratic term did not significantly improve model fit compared to the linear‐only model (*p* = 0.370); therefore, we opted for the more parsimonious linear model for this stage. High intraclass correlation coefficients (0.91 for Stage 1, 0.93 for Stage 2, and 0.93 for Stage 3) indicated substantial variability in average typing fluency between individuals. Conversely, only a small proportion of the variation in typing fluency was attributable to intra‐individual (within‐student) variability, i.e., changes due to learning.

Table 4 presents the LMM parameter estimates for each of the learning stages. In all three stages, the average intercept for typically developing students was substantially higher than that for students with dyslexia, indicating that the latter typed considerably more slowly. In all stages, the parameter *b*_1_ was positive for students with dyslexia, demonstrating clear progress over time. This parameter was significantly higher in their typically developing peers (see rows TD *b*_1_—DD *b*_1_ in Table 4), indicating even faster progress in this group. For students with dyslexia, the parameter *b*_2_ was negative in Stages 1 and 2, reflecting a decelerating learning curve—that is, these students began to approach a plateau in typing fluency during the first two stages. In Stage 1, the deceleration of the learning curve in typically developing students was similar to that of students with dyslexia (see rows TD *b*_2_—DD *b*_2_ in Table 4). However, in Stage 2, the deceleration was significantly more pronounced in typically developing students, suggesting they reached the plateau more quickly than their peers with dyslexia. In Stage 3, which was more challenging than Stages 1 and 2, the ten 10‐min intervals did not appear sufficient for the learning curve to reach a plateau in either group.

**TABLE 4:** Estimates of LMM regression coefficients across the stages of touch‐typing learning.

Parameters	Estimate	SE	df	*t*	*p*	95% CI
Stage 1						
DD *b*_0_	89.37	4.77	36.3	18.75	< 0.001	[80.03, 98.71]
TD *b*_0_	124.52	4.65	36.4	26.75	< 0.001	[115.40, 133.64]
DD *b*_1_	7.68	0.37	33.7	20.61	< 0.001	[6.95, 8.41]
TD *b*_1_—DD *b*_1_	3.10	0.74	33.7	4.17	< 0.001	[1.65, 4.57]
DD *b*_2_	−0.60	0.09	30.7	−6.36	< 0.001	[−0.79, −0.42]
TD *b*_2_—DD *b*_2_	−0.07	0.18	30.7	−0.35	0.726	[−0.44, 0.31]
Stage 2						
DD *b*_0_	103.88	5.46	35.2	19.03	< 0.001	[93.18, 114.57]
TD *b*_0_	151.87	5.33	35.2	28.51	< 0.001	[141.42, 162.31]
DD *b*_1_	4.85	0.30	37.4	16.21	< 0.001	[4.26, 5.43]
TD *b*_1_—DD *b*_1_	2.847	0.60	37.4	4.76	< 0.001	[1.68, 4.02]
DD *b*_2_	−0.36	0.06	38.9	−6.48	< 0.001	[−0.47, −0.25]
TD *b*_2_—DD *b*_2_	−0.26	0.11	38.9	−2.36	0.023	[−0.48, −0.04]
Stage 3						
DD *b*_0_	96.26	6.41	37.2	15.03	< 0.001	[83.71, 108.81]
TD *b*_0_	144.01	6.25	37.2	23.03	< 0.001	[131.75, 156.26]
DD *b*_1_	4.13	0.35	27.5	11.83	< 0.001	[0.45, 4.82]
TD *b*_1_—DD *b*_1_	2.85	0.70	27.5	4.08	< 0.001	[1.48, 4.22]

*Note:* Regression coefficients: *b*_0_ = average fluency in the middle of the stage; *b*_1_ = learning speed; *b*_2_ = learning acceleration. Before estimating the parameters in the models, we excluded outliers in each 10‐min interval for each student group.

Abbreviations: DD, students with developmental dyslexia; TD, typically developing students.

Regarding individual learning curves, we observed a low correlation between parameters *b*_0_ and *b*_1_ across students in Stage 1 (*r* = 0.24), a moderate correlation in Stage 2 (*r* = 0.33) and a high correlation in Stage 3 (*r* = 0.55). These correlations suggest that students with higher typing fluency at the midpoint of a stage tended to progress slightly faster on average than their less fluent peers. Additionally, a strong negative correlation between parameters *b*_0_ and *b*_2_ in Stage 3 (*r* = −0.82) indicated that students with higher mid‐stage typing fluency experienced a greater deceleration in learning over time—in other words, they were closer to reaching their learning plateau.

Next, we examined which abilities predicted students' typing fluency at specific learning stages. Due to the small sample size, additional fixed‐effects covariates were entered into the previously presented LMMs one variable at a time: handwriting fluency, self‐taught typing technique fluency, fluency in a simple finger typing task, and SNAP variables: text reading fluency, non‐word reading fluency, phonological awareness, spelling fluency, verbal working memory, and speed in picture naming. For each learning stage, we thus estimated nine separate models, each including one predictor, and examined the parameter estimate for the interaction between the predictor and *b*_0_. Table 5 summarises the predictors for which these interaction terms reached statistical significance when added to the model. We found that self‐taught typing technique fluency, non‐word reading fluency, and final simple finger typing task fluency were statistically significant predictors of touch‐typing fluency outcomes at all stages of touch‐typing learning.

**TABLE 5:** Statistical significance (*p*) of predictors included in linear mixed models of typing fluency at each of the three stages of touch‐typing learning for both groups of students together.

	1st stage AFJČ	2nd stage ADFJKČ	3rd stage ASDFJKLČ
Predictor			
STT	< 0.001	< 0.001	< 0.001
SNAP NW	0.002	0.002	0.004
FTT	0.004	< 0.001	< 0.001
HW	0.111	0.491	0.253
SNAP RF	0.344	0.300	0.100
SNAP WM	0.313	0.245	0.863
SNAP SN	0.223	0.193	0.375
SNAP SPELL	0.052	0.188	0.259
SNAP PHON	0.157	0.222	0.402

*Note:* The number of participants ranged from 38 to 42, and the number of observations ranged from 256 to 310 across models. Some touch‐typing performance data were missing due to technical difficulties during data collection, as previously reported. In addition, observations identified as outliers were excluded a priori.

Abbreviations: FTT, simple typing task fluency; HW, handwriting fluency; SNAP NW, non‐word reading fluency; SNAP PHON, phonological awareness; SNAP RF, reading fluency; SNAP SN, speed in picture naming; SNAP SPELL, spelling; SNAP WM, verbal working memory; STT, self‐taught typing technique fluency.

To determine the extent to which these predictors jointly explained variance in the final touch‐typing fluency test, we entered them into a multiple linear regression model predicting performance on the final short touch‐typing fluency test administered immediately after the eleventh 10‐min Stage 3 typing interval.

Data from all students were included in the analysis, except for outliers, which were excluded. A forward selection procedure identified the strongest independent predictors, adding only variables that improved model fit. Moderate correlations were observed between all variables and the touch‐typing fluency test result. All key assumptions of linear regression were met: the residuals were approximately normal and homoscedastic, and were linearly related to the predictors. No multicollinearity was detected; the errors were independent (Durbin–Watson statistic = 2.60), and no influential outliers were observed.

The analysis showed that final simple finger‐typing fluency and self‐taught typing technique fluency made significant unique contributions to predicting the result of the touch‐typing fluency test, *F* (2, 28) = 19.82, *p* < 0.001. Simple finger‐typing fluency (*b* = 3.86, 95% CI for *b* = [1.96; 5.76], *p* < 0.001) and self‐taught typing fluency (*b* = 0.54, 95% CI for *b* = [0.18; 0.90], *p* = 0.004) were included in the prediction model, whereas SNAP non‐word reading fluency did not meet the entry criterion. The modest correlation between simple finger‐typing fluency and self‐taught typing technique fluency (*r* = 0.328), together with low collinearity (VIF = 1.12), indicated that they captured largely non‐overlapping variance. Simple finger‐typing fluency uniquely explained 25.5% of the variance in the final typing‐fluency score, whereas self‐taught typing fluency accounted for an additional 14.3%, with the full model explaining 55.6% of the variance (*R* ^2^ = 0.556).

Finally, exploratory correlation analyses were conducted to contextualise the strong predictive value of simple finger‐typing fluency identified in the regression model by examining its associations with dyslexia‐relevant cognitive‐processing capacities assessed with SNAP. The aim was to determine which established dyslexia‐related processing constraints are associated with fine motor typing fluency, thereby clarifying whether its predictive value reflects not only pure motor execution but also broader underlying cognitive demands. From SNAP, speed of picture naming and verbal working memory were selected, as efficient performance on the simple finger‐typing task may (among others) depend on processing speed and the ability to maintain and follow the repetition of the sequence 41,324 in memory. For the whole sample, no association was observed between simple finger‐typing task fluency and speed of picture naming (*r*_s_ = −0.14, *p* = 0.497, *N* = 38), but there was a moderate and statistically significant positive correlation between simple finger‐typing task fluency and verbal working memory (*r*_s_ = 0.40, *p* = 0.013, *N* = 38). Analysed separately for students with and without dyslexia, in the dyslexia group, no association was observed between simple finger‐typing task fluency and speed of naming (*r* = −0.07, *p* = 0.789, *n*_1_ = 19). However, a moderate and statistically significant positive association was found between simple finger‐typing task fluency and working memory (*r*_s_ = 0.54, *p* = 0.020, *n*_1_ = 18). In students without dyslexia, no significant associations were observed between simple finger‐typing task fluency and speed of naming (*r*_s_ = 0.25, *p* = 0.323, *n*_2_ = 18) or between simple finger‐typing task fluency and working memory (*r*_s_ = 0.34, *p* = 0.889, *n*_2_ = 20).

## 5 Discussion

In our previous work (Košir et al. 2024), we reported that students with dyslexia type less fluently than their peers without dyslexia (see the results of the touch‐typing fluency test in Table 2). In the present study, we extended this work by examining differences in the learning trajectories of touch‐typing fluency between the two groups. We found through LMM analysis that the two groups differed not only in their average touch‐typing fluency in different stages of training, but also in the rate and deceleration of improvement over time across the different stages of learning. Students with higher mid‐stage typing fluency tended to progress more quickly and reached the learning plateau sooner. These findings suggest that students with dyslexia may require more time to achieve comparable levels of typing fluency. To our knowledge, no other study has specifically examined the learning curves during touch‐typing acquisition in students with dyslexia. The most comparable study is that of Weigelt‐Marom and Weintraub (2016), who observed similar learning patterns in university students with and without reading and/or writing‐specific learning disorders during touch‐typing acquisition.

In our study, students with dyslexia who exhibited difficulties in language processing tasks—such as lower reading text fluency, non‐word reading fluency, and spelling fluency measured with SNAP tests—also demonstrated reduced handwriting fluency, lower self‐taught typing fluency, lower final touch‐typing fluency, and, according to the LMM analysis in this study, slower progress in touch‐typing fluency compared to their typically developing peers. These findings align with previous research reporting differences in gross and fine motor learning among students with dyslexia (Marchand‐Krynski et al. 2017; Nicolson et al. 2010; Nicolson and Fawcett 2018), and with the difficulties experienced by students with dyslexia when the motor task involved linguistic units, compared to motor tasks without linguistic units (Gabay et al. 2012).

We found that both the learning curve of touch‐typing fluency and the final touch‐typing fluency were significantly associated with performance on the simple finger typing task, prior self‐taught typing fluency, and scores on the SNAP non‐word reading fluency test. Similarly to our findings, Preminger et al. (2004) reported that the speed of one's own (pre‐existing) typing style was significantly correlated with typing speed after learning touch‐typing (*r* = 0.67) and together with the speed and accuracy of reading individual digits from left to right explained 48.3% of the variance in post‐training typing speed. Our results indicated that, in the initial stages of touch‐typing learning, the development of touch‐typing fluency was related to a set of underlying skills involving both motor and linguistic aspects of typing skill development. Multiple regression analysis further showed that performance speed on the simple finger‐typing task and the self‐taught typing task accounted for a substantial proportion of unique variance in touch‐typing fluency outcomes. In contrast, non‐word reading fluency, which primarily indexed language‐based processing, did not explain additional variance. Within the model, only variables incorporating a motor component contributed unique explanatory value; notably, the predictor accounting for the largest proportion of unique variance was also the one most strongly defined by motor execution demands, namely the simple finger‐typing task.

The simple typing task, which accounted for the largest proportion of unique variance, involved the repeated execution of a fixed, simple, one‐handed fine‐motor sequence without visual keyboard guidance or text‐processing demands. Accordingly, task requirements were limited to the maintenance and execution of a stable one‐handed motor sequence, in contrast to touch‐typing, which additionally requires coordination of complex bimanual motor patterns and the temporary processing of phonological and orthographic information presented onscreen. These findings indicate that, during the earliest stages of touch‐typing acquisition limited to the home‐row keys, performance is at least partly associated with simple fine‐motor keyboard execution efficiency. In line with this, early touch‐typing fluency appears to be associated with basic fine‐motor skills involved in simple typing sequences, before the full keyboard is introduced and higher‐level linguistic or cognitive demands increase. Importantly, this pattern of associations does not imply a purely motor‐based explanation rather, it appears to also reflect the specific task demands characteristic of this early learning stage, as well as particular features of the simple typing task itself, which may implicitly capture the contribution of additional capacities supporting sequential task execution. In line with this interpretation, additional exploratory correlational analyses of the simple finger‐typing task incorporating dyslexia‐relevant cognitive measures—specifically rapid automatized naming of pictures as an index of processing speed and verbal working memory—revealed no association with naming speed, whereas a moderate association was observed with working memory in both the full sample and the subgroup of students with dyslexia. Accordingly, individual differences in touch‐typing fluency may be interpreted as reflecting the integration of fine‐motor execution with other capacities inherent to simple finger‐typing performance, particularly those related to maintaining and executing stable action sequences over time (e.g., working memory). Notably, given the speeded and repetitive nature of this task, examining the contribution of attentional resources would also be highly relevant, especially considering that attentional control may pose additional challenges for students with dyslexia; however, such analyses are beyond the scope of the present study.

It remains unclear whether the pattern of associations observed in the present study extends across the full trajectory of touch‐typing acquisition. As learners progress, the relative role of cognitive, linguistic, and motor‐related contributors may vary, particularly as typing moves from isolated word production with a restricted set of keys to sustained continuous text entry involving the full keyboard. Importantly, increasing task complexity over the course of touch‐typing learning may become more demanding for students with dyslexia, particularly as along motor, also linguistic load increases. Language‐related components commonly highlighted in the literature as predictors of fluent keyboarding/typing—such as phonological encoding, orthographic knowledge (Rosenberg‐Adler and Weintraub 2020), and reading speed (Khoury‐Shaheen and Weintraub 2023; Gahshan‐Haddad and Weintraub 2023)—may thus become more influential at later stages of skill development, as later stages of typing typically require continuous reading of text‐level information, which may also affect the rate of fluency improvement and the development of automatization beyond the early phases of touch‐typing acquisition.

Our results also align with evidence showing that, for individuals whose handwriting fluency is compromised by language‐related deficits, typing may not necessarily serve as an effective compensatory strategy (Bisschop et al. 2017; Rosenberg‐Adler and Weintraub 2020). A deeper understanding of how motor, cognitive, and linguistic contributors interact across different learning stages is therefore needed to inform stage‐sensitive instructional and support planning, with the aim of fostering efficient skill acquisition and eventual automatization of touch‐typing.

## 6 Conclusions and Educational Implications

This study found that students with dyslexia displayed a distinct learning curve in touch‐typing fluency. Differences between groups appeared in touch‐typing fluency levels, rate of improvement, and its deceleration as the learning plateau approached. Simple motor typing fluency showed a strong association with early touch‐typing fluency and emerged as the strongest predictor of final touch‐typing fluency. This finding underscores the importance of early motor‐sequential efficiency, while also suggesting that successful performance at this stage reflects the integration of fine‐motor execution with basic cognitive supports inherent to rapid sequential task demands, such as verbal working memory.

While students with dyslexia may not reach the touch‐typing fluency of their peers, encouraging them to achieve their personal best can improve writing efficiency by freeing cognitive resources for content. If touch‐typing remains challenging, alternatives such as speech‐to‐text should be considered.

## 7 Limitations and Future Research Directions

Limitations include a small sample size and the presence of co‐occurring writing or attention difficulties in many students with dyslexia, which may have affected their performance. The groups were not matched on control tasks, and the brief training period captured only the early stages of touch‐typing.

The analyses included only data collected under conditions of comparable instructional support. Data from the end of Stage 3, where assistance began to vary across students and was not systematically monitored or recorded, were not included. To properly assess the effects of such procedural variability, systematic recording of instructional support would have been necessary, which was beyond the scope of the current study. Future research should therefore explicitly track students' practice alongside variability in instructional support and examine learning trajectories under differing support conditions.

A limitation of the study concerns the operationalization of typing fluency, as pauses longer than 3 s were excluded to assess performance during continuous typing, which may have distorted the actual time students spent typing. In addition, only intervals conducted under comparable instructional conditions were included in the analysis due to feasibility constraints in data collection and analysis. This may have limited the representation of fluency across the full course of touch‐typing acquisition. Future research should include all instructional intervals to capture the full range of performance. This should be taken into account when interpreting the findings.

Future research should use larger samples to examine how motor, linguistic, different cognitive, and prior typing skills influence touch‐typing at all stages of learning, with the aim of developing more individualised teaching approaches tailored to students' specific needs. Additionally, future studies should investigate whether group differences persist at advanced stages and how fluent typing supports the writing process in students with dyslexia across different writing tasks.

## Author Contributions

**Janja Košir:** conceptualization, project administration, investigation, resources, writing – original draft, methodology, validation, formal analysis, writing – review and editing. **Andrej Košir:** formal analysis, software, data curation, funding acquisition. **Anja Podlesek:** methodology, formal analysis, supervision, writing – review and editing.

## Funding

This work was supported in part by the Slovenian Research Agency under the research program ICT4QoL—Information and Communications Technologies for Quality of Life, grant number P2‐0246 and Research program No. P5‐0110 (Psychological and Neuroscientific Aspects of Cognition).

## Ethics Statement

With the approval of the Ethics Committee of the Faculty of Education of the University of Ljubljana, all parents and students were informed about the research, the use of the data and voluntary participation and gave their written consent; the students received no compensation.

## Conflicts of Interest

The authors declare no conflicts of interest.

## Data Availability

The raw data are deposited in the Zenodo repository (Košir et al. 2025) and are available upon reasonable request, subject to approval by the corresponding author of this study.

## References

[dys70053-bib-0001] Abecassis, S. , H. Magen , and N. Weintraub . 2023. “Typing Performance and Technique of Higher Education Students With Specific Learning Disorders.” Learning Disabilities Research & Practice 38, no. 2: 119–128. 10.1111/ldrp.12305.

[dys70053-bib-0002] Ahmed, Y. , S. Kent , P. T. Cirino , and M. Keller‐Margulis . 2021. “The Not‐So‐Simple View of Writing in Struggling Readers/Writers.” Reading and Writing Quarterly 38, no. 3: 272–296. 10.1080/10573569.2021.1948374. PMC9246105 35783450

[dys70053-bib-0003] Ainley, J. , and R. Carstens . 2018. Teaching and Learning International Survey (TALIS) 2018 Conceptual Framework (OECD Education Working Papers, No. 187). OECD Publishing.

[dys70053-bib-0004] Alves, R. A. , S. L. Castro , and T. Olive . 2008. “Execution and Pauses in Writing Narratives: Processing Time, Cognitive Effort and Typing Skill.” International Journal of Psychology 43, no. 6: 969–979. 10.1080/00207590701398951. 22022840

[dys70053-bib-0005] Alves, R. A. , and T. Limpo . 2015. “Progress in Written Language Bursts, Pauses, Transcription, and Written Composition Across Schooling.” Scientific Studies of Reading 19, no. 5: 374–391. 10.1080/10888438.2015.1059838.

[dys70053-bib-0006] Barnett, A. L. , V. Connelly , and B. Miller . 2020. “The Interaction of Reading, Spelling, and Handwriting Difficulties With Writing Development.” Journal of Learning Disabilities 53, no. 2: 92–95. 10.1177/0022219419894565 31847693

[dys70053-bib-0007] Barnett, A. L. , and N. Stuart . 2024. “Understanding Typing Skill in Students With Developmental Disorders.” Current Developmental Disorders Reports 11, no. 2: 63–74. 10.1007/s40474-024-00298-8.

[dys70053-bib-0008] Beaton, L. S. 2012. Keyboarding Methodology Instructional Guide for Teachers and Administrators. CTE Resource Center.

[dys70053-bib-0009] Beers, S. F. , T. Mickail , R. Abbott , and V. Berninger . 2017. “Effects of Transcription Ability and Transcription Mode on Translation: Evidence From Written Compositions, Language Bursts and Pauses When Students in Grades 4 to 9, With and Without Persisting Dyslexia or Dysgraphia, Compose by Pen or by Keyboard.” Journal of Writing Research 9, no. 1: 1–25. 10.17239/jowr-2017.09.01.01. PMC6369520 30761210

[dys70053-bib-0010] Biotteau, M. , Y. Chaix , and J. Albaret . 2015. “Procedural Learning and Automatization Process in Children With Developmental Coordination Disorder and/or Developmental Dyslexia.” Human Movement Science 43: 78–89. 10.1016/j.humov.2015.07.005. 26241333

[dys70053-bib-0011] Bisschop, E. , C. Morales , V. Gil , and E. Jiménez‐Suárez . 2017. “Fluency and Accuracy in Alphabet Writing by Keyboarding: A Cross‐Sectional Study in Spanish‐Speaking Children With and Without Learning Disabilities.” Journal of Learning Disabilities 50, no. 5: 534–542. 10.1177/0022219416633865. 27067940

[dys70053-bib-0012] Björnsson, C. S. 1968. Läsbarhet (Lärarbiblioteket). Liber.

[dys70053-bib-0013] Cellini, N. 2017. “Memory Consolidation in Sleep Disorders.” Sleep Medicine Reviews 35: 101–112. 10.1016/j.smrv.2016.09.003. 27765468

[dys70053-bib-0014] Cerni, T. , and R. Job . 2024. “Spelling Processing During Handwriting and Typing and the Role of Reading and Visual‐Motor Skills When Typing Is Less Practiced Than Handwriting.” Reading and Writing 37, no. 1: 205–237. 10.1007/s11145-023-10418-2.

[dys70053-bib-0015] Christensen, C. A. 2004. “Relationship Between Orthographic‐Motor Integration and Computer Use for the Production of Creative and Well‐Structured Written Text.” British Journal of Educational Psychology 74, no. 4: 551–564. 10.1348/0007099042376373. 15530201

[dys70053-bib-0017] Crump, M. J. , and G. D. Logan . 2010. “Warning: This Keyboard Will Deconstruct—The Role of the Keyboard in Skilled Typewriting.” Psychonomic Bulletin & Review 17, no. 3: 394–399. 10.3758/PBR.17.3.394. 20551364

[dys70053-bib-0018] D‐EDK . 2014. “Lehrplan 21.” https://www.lehrplan.ch.

[dys70053-bib-0019] Degen, B. 1997. Typing 1. DZS.

[dys70053-bib-0020] Denckla, M. B. 1985. “Motor Coordination in Children with Dyslexia: Theoretical and Clinical Implications.” In Dyslexia: A Neuroscientific Approach to Clinical Evaluation, edited by F. H. Duffy and N. Geschwind , 187–195. Little Brown.

[dys70053-bib-0021] Dhakal, V. , A. M. Feit , P. O. Kristensson , and A. Oulasvirta . 2018. “Observations on Typing From 136 Million Keystrokes.” In CHI ‘18 Proceedings of the 2018 CHI Conference on Human Factors in Computing Systems ACM, 1–12. Association for Computing Machinery. 10.1145/3173574.3174220.

[dys70053-bib-0022] Donica, D. K. , P. Giroux , and A. Faust . 2018. “Keyboarding Instruction: Comparison of Techniques for Improved Keyboarding Skills in Elementary Students.” Journal of Occupational Therapy, Schools & Early Intervention 11, no. 4: 396–410. 10.1080/19411243.2018.1512067.

[dys70053-bib-0023] Donica, D. K. , P. Giroux , Y. Kim , and S. Branson . 2021. “A Comparison of Two Keyboarding Instruction Methods Over 2 Years for Elementary Students.” Open Journal of Occupational Therapy 9, no. 3: 1–13. 10.15453/2168-6408.1819.

[dys70053-bib-0024] Enders, C. K. , and D. Tofighi . 2007. “Centering Predictor Variables in Cross‐Sectional Multilevel Models: A New Look at an Old Issue.” Psychological Methods 12, no. 2: 121–138. 10.1037/1082-989X.12.2.121. PMID: 17563168. 17563168

[dys70053-bib-0025] Feng, L. , A. Lindner , X. R. Ji , and R. Malatesha Joshi . 2019. “The Roles of Handwriting and Keyboarding in Writing: A Meta‐Analytic Review.” Reading and Writing 32, no. 1: 33–63. 10.1007/s11145-017-9749-x.

[dys70053-bib-0026] Finnish National Agency for Education . 2016. National Core Curriculum for Basic Education 2014.

[dys70053-bib-0028] Gabay, Y. , R. Schiff , and E. Vakil . 2012. “Dissociation Between the Procedural Learning of Letter Names and Motor Sequences in Developmental Dyslexia.” Neuropsychologia 50, no. 10: 2435–2441. 10.1016/j.neuropsychologia.2012.06.014. 22750119

[dys70053-bib-0029] Gahshan‐Haddad, N. , and N. Weintraub . 2023. “Underlying Functions Associated With Keyboarding Performance of Elementary‐School Students.” Scandinavian Journal of Occupational Therapy 30, no. 8: 1415–1423. 10.1080/11038128.2023.2188254. 36927326

[dys70053-bib-0031] Graham, S. , A. A. Aitken , M. Hebert , et al. 2021. “Do Children With Reading Difficulties Experience Writing Difficulties? A Meta‐Analysis.” Journal of Educational Psychology 113, no. 8: 1481–1506. 10.1037/edu0000643.

[dys70053-bib-0033] Hong, J. Y. , E. Gallanter , E. M. Müller‐Oehring , and T. Schulte . 2019. “Phases of Procedural Learning and Memory: Characterisation With Perceptual‐Motor Sequence Tasks.” Journal of Cognitive Psychology 31, no. 5–6: 543–558. 10.1080/20445911.2019.1642897. PMC8048153 33868637

[dys70053-bib-0034] Horne, J. , J. Ferrier , C. Singleton , and C. Read . 2011. “Computerised Assessment of Handwriting and Typing Speed.” Educational and Child Psychology 28, no. 2: 52–62. 10.53841/bpsecp.2011.28.2.52.

[dys70053-bib-0035] Ishitani, Y. , N. Matsuura , M. Hiratani , and M. Hirayama . 2024. “Typing Skill Acquisition in Children With Developmental Disorders, With or Without Developmental Dyslexia.” Learning Disabilities: A Contemporary Journal 22, no. 2: 197–208.

[dys70053-bib-0036] Jung, S. , K. Moeller , E. Klein , and J. Heller . 2021. “Mode Effect: An Issue of Perspective? Writing Mode Differences in a Spelling Assessment in German Children With and Without Developmental Dyslexia.” Dyslexia 27, no. 3: 373–410. 10.1002/dys.1675. 33615629

[dys70053-bib-0037] Khoury‐Shaheen, R. , and N. Weintraub . 2023. “Arabic Handwriting vs. Keyboarding: Performance and Underlying Body Functions Among Elementary‐School Students.” Journal of Occupational Therapy, Schools & Early Intervention 18, no. 1: 185–199. 10.1080/19411243.2023.2215757.

[dys70053-bib-0038] Košak‐Babuder, M. , J. Kormos , M. Ratajczak , and K. Pižorn . 2019. “The Effect of Read‐Aloud Assistance on the Text Comprehension of Dyslexic and Non‐Dyslexic English Language Learners.” Language Testing 36, no. 1: 51–75. 10.1177/0265532218756946.

[dys70053-bib-0072] Košir, J. , A. Košir , A. Podlesek , and M. Košak Babuder . 2024. “Typing Fluencies of 12–13‐Year‐Old Students With Dyslexia and Peers With Typical Development.” Reading & Writing Quarterly 40, no. 6: 600–618. 10.1080/10573569.2024.2304758.

[dys70053-bib-0071] Košir, J. , A. Podlesek , and A. Košir 2025. Typing Fluencies of Dyslexia Students and Peers with Typical Development Dataset (Version 2) [Data set]. Zenodo. 10.5281/zenodo.15240626.

[dys70053-bib-0039] Logan, G. D. , J. E. Ulrich , and D. R. Lindsey . 2016. “Different (Key) Strokes for Different Folks: How Standard and Nonstandard Typists Balance Fitts' Law and Hick's Law.” Journal of Experimental Psychology: Human Perception and Performance 42, no. 12: 2084–2102. 10.1037/xhp0000272. 27748613

[dys70053-bib-0040] Lum, J. A. G. , M. T. Ullman , and G. Conti‐Ramsden . 2013. “Procedural Learning Is Impaired in Dyslexia: Evidence From a Meta‐Analysis of Serial Reaction Time Studies.” Research in Developmental Disabilities 34, no. 10: 3460–3476. 10.1016/j.ridd.2013.07.017. PMC3784964 23920029

[dys70053-bib-0041] Malpique, A. A. , D. Valcan , D. Pino‐Pasternak , S. Ledger , M. Asil , and T. Teo . 2023. “The Keys of Keyboard‐Based Writing: Student and Classroom‐Level Predictors of Keyboard‐Based Writing in Early Primary.” Contemporary Educational Psychology 75: 102227. 10.1016/j.cedpsych.2023.102227.

[dys70053-bib-0042] Mangen, A. , and J. L. Velay . 2010. “Digitizing Literacy: Reflections on the Haptics of Writing.” Advances in Haptics 1, no. 3: 86–401. 10.5772/8710.

[dys70053-bib-0043] Marchand‐Krynski, M. È. , O. Morin‐Moncet , A. M. Bélanger , M. H. Beauchamp , and G. Leonard . 2017. “Shared and Differentiated Motor Skill Impairments in Children With Dyslexia and/or Attention Deficit Disorder: From Simple to Complex Sequential Coordination.” PLoS One 12, no. 5: e0177490. 10.1371/journal.pone.0177490. PMC5438138 28542319

[dys70053-bib-0045] Nicolson, R. I. , and A. J. Fawcett . 1990. “Automaticity: A New Framework for Dyslexia Research?” Cognition 35, no. 2: 159–182. 10.1016/0010-0277(90)90013-A. 2354611

[dys70053-bib-0046] Nicolson, R. I. , and A. J. Fawcett . 2000. “Long‐Term Learning in Dyslexic Children.” European Journal of Cognitive Psychology 12, no. 3: 357–393. 10.1080/09541440050114552.

[dys70053-bib-0047] Nicolson, R. I. , and A. J. Fawcett . 2018. “Procedural Learning, Dyslexia and Delayed Neural Commitment.” In Reading and Dyslexia – From Basic Functions to Higher Order Cognition, edited by T. Lachmann and T. Weis , 235–269. Springer.

[dys70053-bib-0048] Nicolson, R. I. , A. J. Fawcett , R. L. Brookes , and J. Needle . 2010. “Procedural Learning and Dyslexia.” Dyslexia: An International Journal of Research and Practice 212: 194–212. 10.1002/dys.408. 20680991

[dys70053-bib-0049] Pinet, S. , C. Zielinski , F. X. Alario , and M. Longcamp . 2022. “Typing Expertise in a Large Student Population.” Cognitive Research: Principles and Implications 7, no. 1: 77. 10.1186/s41235-022-00424-3. PMC9356123 35930064

[dys70053-bib-0050] Poole, D. M. , and M. K. Preciado . 2016. “Touch Typing Instruction: Elementary Teachers' Beliefs and Practices.” Computers & Education 102, no. 1: 1–14. 10.1016/j.compedu.2016.06.008.

[dys70053-bib-0051] Preminger, F. , P. L. Weiss , and N. Weintraub . 2004. “Predicting Occupational Performance: Handwriting Versus Keyboarding.” American Journal of Occupational Therapy 58, no. 2: 193–201. 10.5014/ajot.58.2.193. 15068155

[dys70053-bib-0053] Rosenberg‐Adler, T. , and N. Weintraub . 2020. “Keyboarding Difficulties: Frequency and Characteristics Among Higher Education Students With Handwriting Difficulties.” Learning Disabilities Research and Practice 35, no. 2: 82–88. 10.1111/ldrp.12220.

[dys70053-bib-0055] Smith‐Spark, J. H. , and R. Gordon . 2022. “Automaticity and Executive Abilities in Developmental Dyslexia: A Theoretical Review.” Brain Sciences 12, no. 4: 446. 10.3390/brainsci12040446 PMC9030885 35447978

[dys70053-bib-0057] Stevenson, N. C. , and C. Just . 2014. “In Early Education, Why Teach Handwriting Before Keyboarding?” Early Childhood Education Journal 42, no. 1: 49–56. 10.1007/s10643-012-0565-2.

[dys70053-bib-0069] Thompson, R. , S. Tanimoto , R. Abbott , et al. 2017. “Relationships Between Language Input and Letter Output Modes in Writing Notes and Summaries for Students in Grades 4 to 9 With Persisting Writing Disabilities.” Assistive Technology: The Official Journal of RESNA 29, no. 3: 131–139. 10.1080/10400435.2016.1199066. PMC5291827 27434553

[dys70053-bib-0058] Van Heuverswyn, E. , C. Gosse , and M. Van Reybroeck . 2024. “Handwriting Difficulties in Children With Dyslexia: Poorer Legibility in Dictation and Alphabet Tasks, Slowness in the Alphabet Task.” Dyslexia 30, no. 2: e1767. 10.1002/dys.1767 38684454

[dys70053-bib-0059] Van Waes, L. , M. Leijten , E. Lindgren , and Å. Wengelin . 2017. “Keystroke Logging in Writing Research: Analyzing Online Writing Processes.” In Handbook of Writing Research, edited by A. MacArthur , S. Graham , and J. Fitzgerald , 2nd ed., 410–426. Guilford Press.

[dys70053-bib-0060] Van Weerdenburg, M. , M. Tesselhof , and H. van der Meijden . 2018. “Touch‐Typing for Better Spelling and Narrative‐Writing Skills on the Computer.” Journal of Computer Assisted Learning 35, no. 1: 143–152. 10.1111/jcal.12323.

[dys70053-bib-0061] Weedon, C. , and G. Reid . 2008. Special Needs Assessment Profile‐Specific Learning Difficulties *v*. 3 (SNAP‐SpLD).

[dys70053-bib-0062] Weigelt‐Marom, H. , and N. Weintraub . 2015. “The Effect of a Touch‐Typing Program on Keyboarding Skills of Higher Education Students With and Without Learning Disabilities.” Research in Developmental Disabilities 47: 208–217. 10.1016/j.ridd.2015.09.014. 26447834

[dys70053-bib-0063] Weigelt‐Marom, H. , and N. Weintraub . 2016. “Touch‐Typing Acquisition Process: A Comparison Between Students With and Without Learning Disabilities.” In Learning in the Technological Era, Proceedings of the 11th Chais Conference for the Study of Innovation and Technologies, edited by Y. Eshet‐Alkalai , I. Balu , A. Caspi , N. Geri , Y. Kalman , and V. Sibler‐Varod , 82–89. Open University of Israel. https://www.openu.ac.il/innovation/chais2016/c1_2.pdf.

[dys70053-bib-0064] Weigelt‐Marom, H. , and N. Weintraub . 2018. “Keyboarding Versus Handwriting Speed of Higher Education Students With and Without Learning Disabilities: Does Touch‐Typing Assist in Narrowing the Gap?” Computers & Education 117: 132–140. 10.1016/j.compedu.2017.10.008.

[dys70053-bib-0070] Wengelin, Å. , R. Johansson , and V. Johansson . 2014. “Expressive Writing in Swedish 15‐Year‐Olds With Reading and Writing Difficulties.” In Writing Development in Children With Hearing Loss, Dyslexia, or Oral Language Problems: Implications for Assessment and Instruction. Oxford Academic. 10.1093/acprof:oso/9780199827282.003.0018.

[dys70053-bib-0065] Wolff, P. H. 2002. “Timing Precision and Rhythm in Developmental Dyslexia.” Reading and Writing 15: 179–206. 10.1023/A:1013880723925.

[dys70053-bib-0068] Yap, R. L. , and A. van der Leij . 1994. “Testing the Automatization Deficit Hypothesis of Dyslexia via a Dual‐Task Paradigm.” Journal of Learning Disabilities 27, no. 10: 660–665. 10.1177/002221949402701006. 7844482

